# Computational modeling of heart failure in microgravity transitions

**DOI:** 10.3389/fphys.2024.1351985

**Published:** 2024-06-21

**Authors:** Stefan L. Wilson, Klaus-Martin Schulte, Anne Steins, Russell L. Gruen, Emma M. Tucker, Lex M. van Loon

**Affiliations:** College of Health and Medicine, Australian National University, Canberra, ACT, Australia

**Keywords:** space medicine, cardiovascular, mathematical model (MM), digital twin, physiology

## Abstract

The space tourism industry is growing due to advances in rocket technology. Privatised space travel exposes non-professional astronauts with health profiles comprising underlying conditions to microgravity. Prior research has typically focused on the effects of microgravity on human physiology in healthy astronauts, and little is known how the effects of microgravity may play out in the pathophysiology of underlying medical conditions, such as heart failure. This study used an established, controlled lumped mathematical model of the cardiopulmonary system to simulate the effects of entry into microgravity in the setting of heart failure with both, reduced and preserved ejection fraction. We find that exposure to microgravity eventuates an increased cardiac output, and in patients with heart failure there is an unwanted increase in left atrial pressure, indicating an elevated risk for development of pulmonary oedema. This model gives insight into the risks of space flight for people with heart failure, and the impact this may have on mission success in space tourism.

## 1 Introduction

Space medicine has traditionally focused on studying the effects of microgravity exposure on healthy, professional astronauts. However, with the advent of low-cost, reusable rocketry, space travel is becoming increasingly accessible to the public. This shift in accessibility raises concerns as individuals with underlying medical conditions may be exposed to a microgravity environment. Commercial spaceships typically subject passengers to a period of hypergravity (up to ∼3G) before experiencing an almost instantaneous transition to near microgravity (∼0G) (Jackson, 2017). Such transitions result in cardiovascular hemodynamic changes; at longer duration they cause fluid redistribution from the lower half to the top half of the body, commonly referred to as ‘puffy face bird leg’ syndrome (Williams et al., 2009). This fluid redistribution leads to reduced venous pooling in the lower limbs and increased venous pressures in upper-body compartments (Leach et al., 1996; Schneider et al., 2016).

The target demographic of privatized space tourism significantly differs from that of professional astronauts, as it is likely to prioritize income over fitness. Consequently, understanding the behaviour of chronic health conditions - such as heart failure-in microgravity transitions becomes crucial to ensure mission safety in space tourism. This study focuses on heart failure due to its impact on the cardiovascular system and its prevalence among the global population. Heart failure is a common scenario, affecting some 100 million people worldwide (Savarese et al., 2022), with a prevalence of 1%–5% in the age group over 18 years (Sahle et al., 2016) and an incidence that increases with age, resulting in an overall lifetime risk of 1 in 5 for both men and women (Lloyd-Jones et al., 2002).

Heart failure can be classified based on the left ventricular ejection fraction (LVEF). Heart Failure with reduced Ejection Fraction (HFrEF) is characterized by LVEF ≤40%, while Heart Failure with preserved Ejection Fraction (HFpEF) has LVEF >40%. Although these classification groups comprise heterogeneous disorders, certain fundamental cardiovascular and hemodynamic properties within each group can be used to model effects. HFrEF involves *systolic dysfunction*, characterized by eccentric left ventricular remodeling, left ventricular dilation, and a decrease in systolic left ventricular elastance (i.e., myocardial contraction). On the other hand, HFpEF is characterized by *diastolic dysfunction*, involving concentric left ventricular remodeling, increased diastolic left ventricular elastance (i.e., myocardial relaxation), and impaired left ventricular filling (Zile et al., 2004; Simmonds et al., 2020).

To our knowledge, no studies exist on the effects of microgravity specifically on individuals with heart failure. While one recent study observed a decrease in heart failure biomarkers during parabolic flight, this research was limited to healthy adults, and up to now no investigations have explored the cardiovascular hemodynamic effects of microgravity transitions in people with heart failure (Jirak et al., 2020). Given the absence of available data on patients with heart failure in microgravity, the use of mathematical simulations is deemed appropriate to study this topic. Mathematical models have proven valuable in space medicine for predicting physiological changes that occur after exposure to microgravity (Wieling et al., 2002; Mohammadyari et al., 2021; van Loon et al., 2022).

Therefore, the primary aim of this study is to simulate the effects and assess the risks associated with microgravity transitions for individuals with heart failure.

## 2 Methods

### 2.1 Base model

**FIGURE 1:**
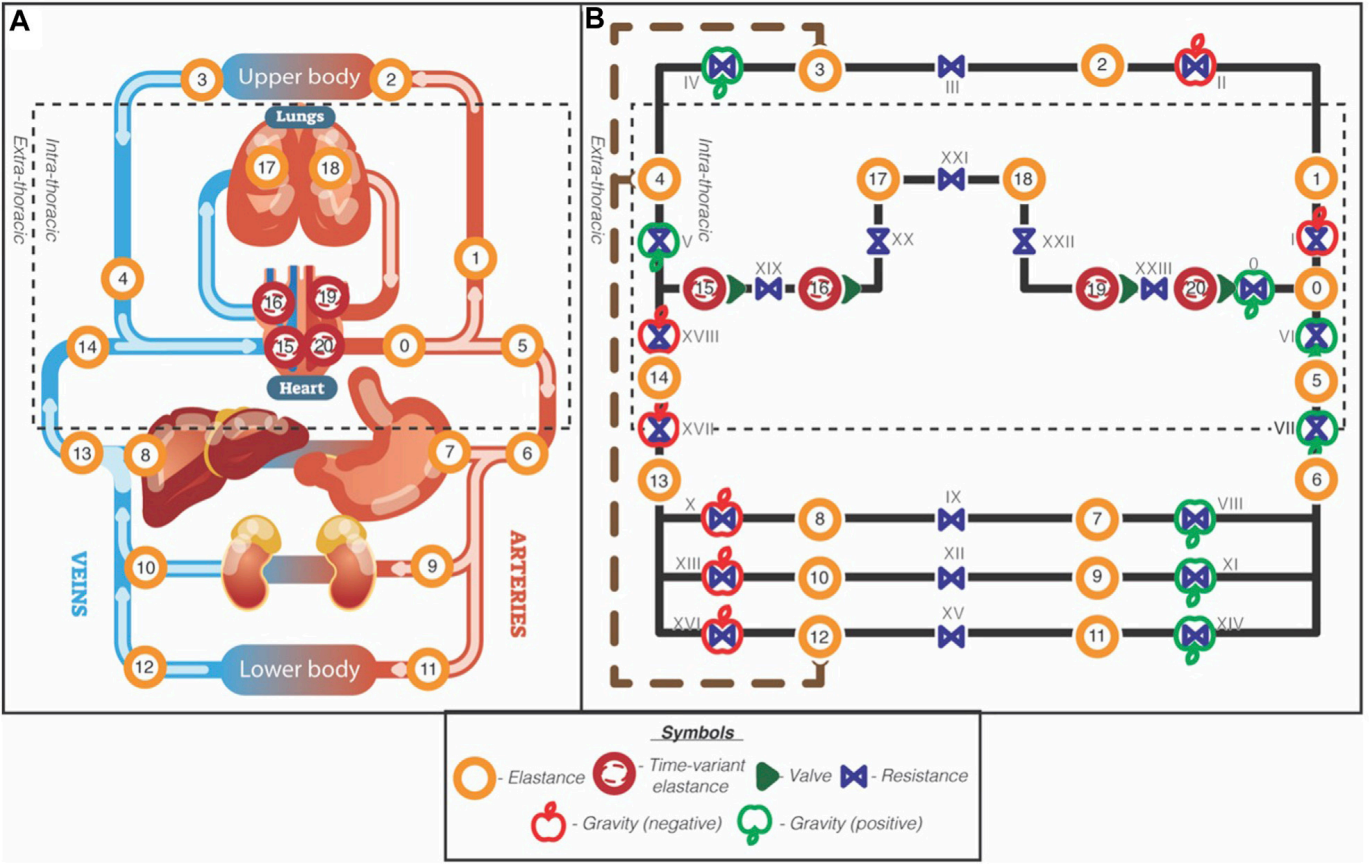
Schematic overview of the 21-compartment cardiovascular model. Reproduced from van Loon et al., 2022, licensed under CC BY 4.0 International. Left panel **(A)**: Anatomic model. Dashed square indicates the intrathoracic pressure. Right panel **(B)**: Hydraulic circuit model. The orange circles with numbers are elastic elements with a pre and post resistance in blue and annotated with Roman numbers. The cardiac compartments are illustrated with red circles and represent time-variant elastances, together with their valves (green single triangles). The dashed rectangle outlines the intrathoracic compartments, and the brown wide-dashed line with round arrowheads indicates the lymphatic flow from the lower and upper body to the super vena cava. The green and red apple indicate gravity and its direction (green = added, red = subtracted).

This project built upon the work of van Loon, Steins (van Loon et al., 2022), who established a controlled 21-compartment mathematical model of the cardiovascular system. In short, the model consists of four time-varying elastic heart compartments, 5 intra-thoracic compartments, two upper-body compartments, and eight lower-body compartments. The model uses Poiseuille’s Law to calculate pressure in each compartment based on the volume, and Ohm’s aw to describe the fluid mechanics between compartments. Each compartment is characterised with an inflow/outflow resistance, elastance and an unstressed volume. The heart compartments were modelled using time-varying elastances to simulate the heart as a pressure source. The conceptual model is presented in Figure 1. An extensive model description and parameter allocation of this base model can be found here (van Loon et al., 2022).

**Table:** 

*0: Ascending aorta*	*7: Renal arteries*	*14: Thoracic inferior vena cava*
*1: Brachiocephalic arteries*	*8: Renal veins*	*15: Right atrium*
*2: Upper body arteries*	*9: Splanchnic arteries*	*16: Right ventricle*
*3: Upper body veins*	*10: Splanchnic veins*	*17: Pulmonary arteries*
*4: Superior vena cava*	*11: Lower body arteries*	*18: Pulmonary veins*
*5: Thoracic aorta*	*12: Lower body veins*	*19: Left atrium*
*6: Abdominal aorta*	*13: Abdominal veins*	*20: Left ventricle*

### 2.2 Parameter estimation

Based on the pathophysiology of HFrEF and HFpEF as previously described, relevant parameters associated with HFrEF (such as left ventricular maximum elastance (LV max elastance, mmHg/mL) and left ventricular end-diastolic volume (LVEDV, ml)) and parameters related to HFpEF (such as left ventricular minimum elastance (LV min elastance, mmHg/mL) and left atrial unstressed volume (mL)) were obtained from the literature. The weighted average of these parameters was calculated based on the participant sample size in each study. This process was conducted separately for HFrEF (Yip et al., 2011; Schwartzenberg et al., 2012; Warriner et al., 2014; Obokata et al., 2017; Charman et al., 2022) and HFpEF (Lam et al., 2009; Penicka et al., 2010; Ohtani et al., 2012; Schwartzenberg et al., 2012; Abudiab et al., 2013; Rommel et al., 2016; Obokata et al., 2017). Detailed parameter information for heart failure can be found in Table 1.

**TABLE 1:** Parameters of a healthy astronaut (“normal”) and HFrEF and HFpEF. “Normal” literature value ranges in brackets for comparison (Lam et al., 2009; Penicka et al., 2010; Ohtani et al., 2012; Abudiab et al., 2013; Rommel et al., 2016; Ohno, 2018).

Parameter values	Normal	HFrEF	HFpEF
LV Emax (mm Hg/mL)	2.50 (2.23–3.7)	0.99	2.50
LV End systolic volume (mL)	65.9 (23.7–71.8)	184.2	61.1
LV Emin (mm Hg/mL)	0.10 (0.08–0.17)	0.10	0.21
LA End Systolic Volume (mL)	32.1 (40–70)	42.4	53.6

LV, left ventricle; LV, Emax = left ventricular max (systolic) elastance (aka Ees), LV, Emin = left ventricular minimal (diastolic) elastance, LA, left atrial.

To validate the parameters used in simulating both types of heart failure, the simulation results were compared against established hemodynamic properties of heart failure under normal gravitational conditions. The pressure-volume (PV) loops of heart failure within the model were validated against existing literature under conditions of supine position in normal gravity to ensure consistency.

In individuals with heart failure, the risk of developing acute pulmonary oedema is associated with left atrial pressure (LAP) (Drake and Doursout, 2002). Therefore, LAP was one of the key output parameters analysed in this study.

In the construction of our model, we incorporated data reflecting a diverse range of astronaut demographics. The subjects encompassed 33 individuals from various space missions. Among these, 14 crew members, including 11 men and three women, had a mean age at launch of 40 years, ranging from 31 to 50 years. Their average height was reported as 177 cm, with a range from 158 to 189 cm, and an average weight of 77.3 kg, ranging from 58.2 to 95.2 kg (Fritsch et al., 1992; Buckey et al., 1996; Cooke et al., 2000). To simulate the transition into microgravity, the model employed a gradual change from a ‘standing’ position (1G) to a ‘supine’ position (0G), which is a method that has been used previously in the literature in the computational modeling of microgravity transitions (Gerber et al., 2018; van Loon et al., 2022) During this transition the unstressed volumes of specific fluid compartments are altered to mimic the fluid redistribution observed in space, as described in relevant literature. The specific alterations in unstressed volume can be found in the supplemental tables of van Loon, Steins (van Loon et al., 2022).

The simulated transition into microgravity was performed over a period of 10 s, aiming to replicate the nearly instantaneous shift experienced during actual spaceflight (Jackson, 2017). The initiation of microgravity simulation occurred at 125 s. Data outputs before entry into microgravity were recorded at t = 80 s in the supine position, while the outputs after entry into microgravity were collected at t = 225 s. These time intervals were chosen to allow sufficient stabilization of the model after the simulation initiation and the microgravity transition.

### 2.3 Parameter estimation for HFrEF

Warriner, Brown (Warriner et al., 2014) performed a literature review of PV loops in HFrEF which found that stage C HFrEF (according to American Heart Association (AHA)/American College of Cardiology (ACC) guidelines) was the earliest stage that showed statistically significant changes in parameters of heart failure compared to a healthy population. From this review, it was decided that modeling stage C HFrEF—*i.e.*, a patient with prior or current symptoms, but not experiencing acute refractory heart failure—would be appropriate for this study. The statistically significant cardiovascular parameters that could be modelled were the LV max elastance (mm Hg/mL), and the LV end-systolic and diastolic volume (mL). Both values were substituted into the paper as a change from the data of a healthy astronaut (van Loon et al., 2022). When done so, the proportionally substituted value for Emax was almost identical to the figures from the literature. The change in LV volume from the literature was substituted as a proportional change in LV unstressed volume. When done so, the proportionally substituted value for Emax was almost identical to the figures from the literature (Yip et al., 2011; Schwartzenberg et al., 2012; Warriner et al., 2014; Obokata et al., 2017; Charman et al., 2022). Refer to Table 1 for details of parameter estimates.

The average patient from which these parameters were estimated is a 58 year old male, with a variety of comorbidities and treatment regimes.

### 2.4 Parameter Estimation for HFpEF

As established by the literature, HFpEF is characterised by an increase in LV diastolic elastance and dilation of the left atrium The LV diastolic elastance was increased proportionally—by a factor of 2—compared to the LV diastolic elastance of healthy astronaut as defined by van Loon, Steins (van Loon et al., 2022), based upon values from the literature (Penicka et al., 2010; Kasner et al., 2015). The left atrial end-diastolic volume was increased proportionally by a factor of ∼1.5 in the same manner (Table 1).

The average HFpEF patient from which these parameters are estimated is a 70 year old female undergoing treatment.

### 2.5 Countermeasures

We hypothesised that pharmacological countermeasures used in heart failure could potentially increase the safety of people with heart failure entering microgravity. Diuretics are used to effectively decrease total blood volume, and are used to treat heart failure (Casu and Merella, 2015). Freis (Freis, 1983), found that thiazide diuretics caused a decrease in plasma volume by ∼15%. As plasma volume is 60% of total blood volume (van Loon et al., 2022), a countermeasure simulation was run with a decrease in total blood volume of 9%.

This study altered cardiac parameters from individuals undergoing heart failure treatment, but without modelling the exact effects of a particular treatment regimen. The countermeasure simulation assumes that the patient was not already volume depleted through diuretics.

## 3 Results

### 3.1 Validating heart failure in the computational model

**FIGURE 2:**
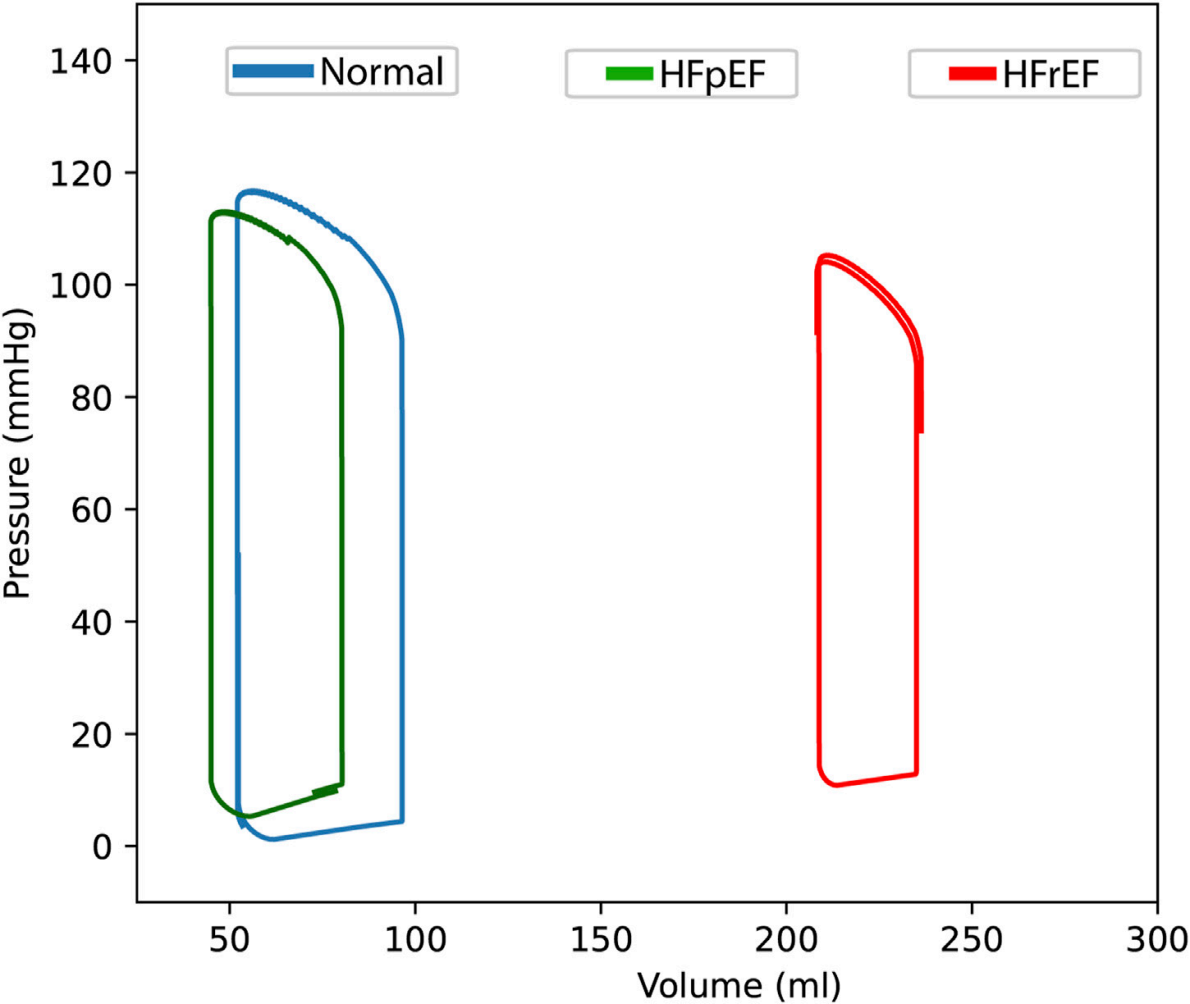
Pressure-volume loops of the left intraventricular compartment. A healthy astronaut (‘normal’, blue line) to heart failure with reduced (HFrEF, red line) and preserved (HFpEF, green line) ejection fraction under Earth’s gravitational conditions (1G) in the supine position (t = 80 s).

The PV loop generated from the mathematical model (Figure 2) using the estimated parameters for HFrEF showed characteristics that are consistent in the literature with PV loops of chronic ischaemic cardiomyopathy (Warriner et al., 2014; Ohno, 2018; Bastos et al., 2019; Jain and Borlaug, 2020; Charman et al., 2022). Refer to Tables 2-4 for detailed parameter outputs.

**TABLE 2:** Simulation output haemodynamic parameters comparing a healthy astronaut (‘normal’) to HFrEF and HFpEF under Earth’s gravitational conditions (1G) in the supine position (t = 80 s). Literature value ranges for comparison (Lam et al., 2009; Penicka et al., 2010; Ohtani et al., 2012; Abudiab et al., 2013; Rommel et al., 2016; Ohno, 2018).

Outputs	Normal simulation output with literature range	HFrEF simulation output with literature range	HFpEF simulation output with literature range
CO (L/min)	5.2 (5.3–6.3)	5.1 (3.1–6)	5.3 (2.8–5.4)
HR (Bpm)	73 (64–75)	77 (70–80)	76 (67–71)
MAP (mm Hg)	97 (80–105)	97 (80–104)	97 (95–104)
LAP (mm Hg)	10 (7–10)	17 (25)	19 (16–23)
SAP (mm Hg)	126 (117–149)	123 (113–143)	122 (125–166)
DAP (mm Hg)	82 (70–80)	83 (73–83)	84 (68–80)
LVEDV (mL)	140 (95–139)	249 (159–237)	126 (107.4–140)
LVESV (mL)	66 (31–68)	184 (112–166)	61 (38–67)
SV (mL)	71 (50–100)	66 (43–78)	69 (50–92.6)
EF (%)	51 (54–67.6)	27 (22–32)	55 (58–65)
Ea (mm Hg/mL)	1.79 (1.5–1.7)	1.87 (1.7–2.8)	1.8 (1.5–2.0)
Ea/Ees	0.72 (0.5–0.6)	1.89 (2.5–3.3)	0.71 (0.6–0.8)

CO, cardiac output; HR, heart rate; MAP = mean arterial pressure; LAP, left atrial pressure; SAP = systolic arterial pressure; DAP, diastolic arterial pressure; LVEDV, left ventricular end diastolic volume; LVESV, left ventricular end systolic volume; SV, stroke volume; EF, ejection fraction, Ea = arterial elastance, Ees = left ventricular end systolic elastance.

**TABLE 3:** Simulation parameter outputs under different conditions.

	Normal			HFrEF			HFpEF		
Outputs	Pre	Post	Δ	Pre	Post	Δ	Pre	Post	Δ
CO (L/min)	4.2	5.4	1.2	3.0	4.5	1.4	3.5	5.1	1.7
HR (Bpm)	86	71	−15	109.7	80.8	−29.0	96.2	76.3	−19.9
MAP (mm Hg)	94.4	96.6	2.2	88.8	95.8	7.0	91.9	96.8	4.9
LAP (mm Hg)	5.6	11.3	5.7	15.2	22.1	6.9	9.2	18.7	9.5
RAP (mm Hg)	0.26	3.0	2.73	−0.7	1.9	2.6	−0.2	2.4	2.7
SAP (mm Hg)	114.8	127.9	13.1	102.7	119.0	16.3	107.7	121.9	14.2
DAP (mm Hg)	84.2	81.0	−3.2	81.9	84.2	2.4	84.0	84.3	0.3
LVEDV (mL)	100.8	147.3	46.5	238.9	298.5	59.6	79.6	122.5	42.9
LVESV (mL)	52.0	69.0	17.0	208.0	242.4	34.5	41.3	59.2	17.9
SV (mL)	45.1	76.4	31.3	27.4	55.1	27.7	36.2	67.5	31.3
EF (%)	44.8	51.9	7.1	11	18	7	45	55	10
Ea (mm Hg/mL)	2.50	1.6	−.96	3.9	2.2	−1.7	3.1	1.8	−1.3
Ea/Ees	1.00	0.66	−0.34	5.9	3.3	−2.6	1.1	0.6	−0.4

Pre = before entry into microgravity (standing, 1G), post = after entry into microgravity (supine, 0G), CO, cardiac output; HR, heart rate; MAP = mean arterial pressure; LAP, left atrial pressure; RAP = right atrial pressure; SAP, systolic arterial pressure; DAP = diastolic arterial pressure; LVEDV, left ventricular end diastolic volume; LVESV, left ventricular end systolic volume; SV, stroke volume; EF, ejection fraction, Ea = arterial elastance, Ees = left ventricular end systolic elastance.

**TABLE 4:** Simulation parameter outputs of heart failure with and without countermeasures.

		HFrEF			HFpEF		
		Pre	Post	Δ	Pre	Post	Δ
Without Countermeasure	CO (L/min)	3	4.5	1.4	3.5	5.1	1.7
	LAP (mm Hg)	15.2	22.1	6.9	9.2	18.7	9.5
With Countermeasure	CO (L/min)	2.64	3.9	1.3	2.8	4.2	1.5
	LAP (mm Hg)	12.5	19.5	7.0	5	15	10.1

Pre = before entry into microgravity, Post = after entry into microgravity, CO, cardiac output; LAP = left atrial pressure, Countermeasure = simulation with 9% less total blood volume.

The right shift of the PV loop of HFrEF, with volumes ∼250 mL and the ejection fraction of ∼27% are all consistent with expected values. The left atrial pressure (LAP) is also increased in a manner consistent with the literature (Drake and Doursout, 2002; Miller Wayne et al., 2013).

Ea/Ees is the ventricular-vascular coupling, a measure of heart contractility coupled with arterial elastance. This was also calculated in HFrEF according to Antonini-Canterin, Poli (Antonini-Canterin et al., 2013) and was shown to be increased in HFrEF compared to normal and HFpEF, which is consistent with the literature (Ohno, 2018; Monge García and Santos, 2020).

**FIGURE 3:**
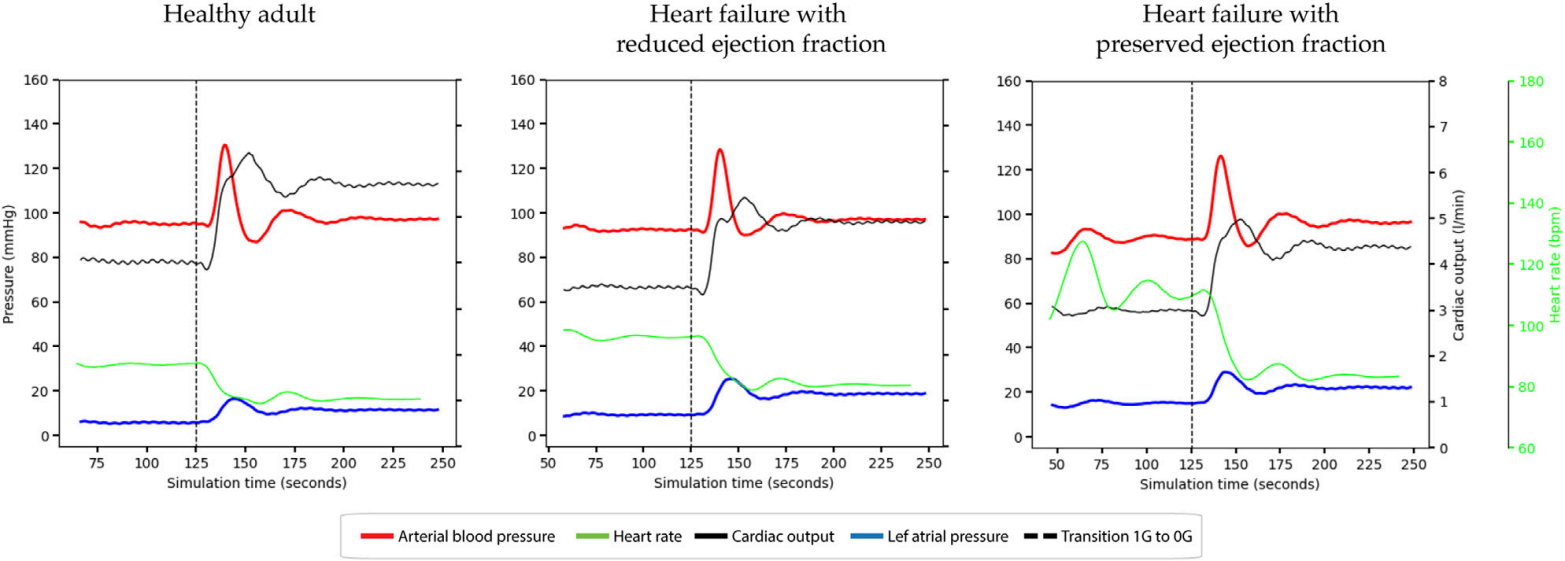
Haemodynamic responses occurring during entry into microgravity under different conditions. *Top panel*; healthy adult. *Middle panel*: Heart failure with reduced ejection fraction (HFrEF). *Bottom panel*: Heart failure with preserved ejection fraction (HFpEF). Line colours: dashed line = start microgravity transition, red = mean arterial blood pressure, dashed green = heart rate, black = cardiac output, and blue = left atrial pressure.

The output from the model after estimating the parameters for HFpEF shows an ejection fraction like that of the healthy astronaut. The end diastolic pressure and the end diastolic pressure-volume relationship (EDPVR) is increased in HFpEF, which can be seen graphically (Figure 3). Left atrial pressure is also increased up to ∼19 mmHg. These findings are all consistent with values from the literature, indicating that the model can simulate some key hemodynamic parameters of HFpEF (Lam et al., 2009; Penicka et al., 2010; Ohtani et al., 2012; Abudiab et al., 2013; Rommel et al., 2016; Ohno, 2018).

### 3.2 Heart failure in the model upon entry into microgravity

Upon entry into microgravity all simulations exhibited the expected increase in cardiac output (CO), with the highest increase observed in individuals with a healthy heart, followed by those with HFpEF and HFrEF. The simulations successfully achieved appropriate stabilization after the fluid redistribution that occurs during the transition into microgravity.

In response to the elevated CO, heart rate (HR) decreased due to autonomic reflexes aimed at conserving cardiac output, as visually depicted in the graphs. Mean arterial pressure (MAP) and diastolic arterial pressure (DAP) remained conserved, indicating overall cardiac and hemodynamic stability across all simulations.

The simulations showed an increase in right atrial pressure (RAP) of approximately 4 mm Hg upon entry into microgravity, which fell within the normal limits previously reported (Beigel et al., 2013). Left atrial pressure (LAP) demonstrated an increase after entering microgravity in all simulations. HFrEF and HFpEF patients demonstrated increases in LAP of 6.9 mm Hg, and 9.5 mm Hg respectively, compared to the healthy adult which increased by 5.7 mm Hg.

During the transition period, LAP exhibited a spike before the model’s autonomic functions could stabilize. This spike caused LAP to reach approximately 14 mm Hg in the healthy simulation, while in the HFrEF and HFpEF simulations, LAP peaked at around 30 mm Hg. These findings highlight the dynamics of LAP during the transition into microgravity, underscoring the physiological changes experienced by individuals with heart failure.

### 3.3 Heart failure upon entry into microgravity with countermeasures

The countermeasure—a decrease in blood volume by 9% - reduces the overall CO and LAP in HFrEF and HFpEF. However, during the microgravity transition both CO and LAP increase in both types of heart failure, though the post-transitional values are reduced compared to non-countermeasure. In HFrEF, CO is reduced pre-flight to 2.64 L/min.

## 4 Discussion

The key findings of this study are as follows: 1) the developed model successfully simulated the fundamental properties of heart failure. 2) Upon entry into microgravity, left atrial pressure (LAP) increased in all simulations, surpassing a clinically significant threshold associated with a serious risk of pulmonary oedema in individuals with heart failure.

### 4.1 Elevated LAP as a risk factor in microgravity entry

The simulations demonstrated a proportional increase in cardiac output in response to microgravity entry, initially suggesting a potential protective effect against heart failure. In all simulations, LAP increased by a similar magnitude after the transition. As patients with heart failure already had elevated LAP under normal gravity (1G) conditions, the post-transitional LAP levels approached values associated with pulmonary oedema. Specifically, HFrEF exhibited a post-transitional LAP of 22 mmHg, while HFpEF went as high as 19 mmHg. Note that a moderately elevated LAP (<25 mmHg) is associated with long-term pulmonary phase changes (Drake and Doursout, 2002). Therefore, even if an individual’s LAP remains below the threshold for acute pulmonary oedema, prolonged exposure to increased LAP does pose a concern.

For the purposes of this study, it is assumed that the post-transitional LAP will be maintained throughout the duration of spaceflight. Therefore, an increase in risk for pulmonary oedema is assumed to be maintained during the entirety of the patient’s time in microgravity. During the transition period, there was a spike in LAP before the autonomic system could compensate. However, this spike is of lesser concern compared to the average post-transitional LAP, as acute pulmonary oedema develops over minutes to hours, whereas the LAP spike occurs for approximately 25 s before the autonomic system establishes a new baseline. Furthermore, literature review suggests no evidence of pulmonary oedema occurring in healthy astronauts upon entry into microgravity, despite inconclusive indications of microgravity as a potential risk factor for its development (Prisk, 2019).

An analogous real-world example of the cardiovascular effects of microgravity transition are those experienced by patients with heart failure undergoing immersion in water. This is analogous due to patients being in a supine position with volume re-distribution whilst immersed—*i.e.*, the two key changes modelled for microgravity simulation (Shah et al., 2019). Two studies found results that were consistent with the simulation. Patients with heart failure had an increase in CO and LAP that was associated with supine immersion in water (Meyer and Bücking, 2004; Shah et al., 2019), indicating a real-world consistency in the cardiovascular parameters of the model. A significant recommendation arising from this study is that individuals with heart failure entering a microgravity environment are at risk of developing pulmonary oedema.

### 4.2 Enhancing safety through countermeasures

The results of countermeasure simulations indicate that a pre-flight reduction in blood volume by approximately 9% using diuretics could sufficiently decrease LAP, reducing the risk of pulmonary oedema in patients with heart failure upon entering microgravity. The countermeasure reduces both pre-flight and post-flight LAP, potentially mitigating the risk of LAP reaching the critical threshold of 25 mmHg (Drake and Doursout, 2002) leading to acute pulmonary oedema. However, the reduction in total blood volume also led to a decrease in pre-flight cardiac output for HFrEF, reaching a cardiac index of 2.01, which is approaching the threshold for risk of developing cardiogenic shock (<1.8 L/min/m^2^) (Verbraecken et al., 2006; Reynolds and Hochman, 2008). Therefore, while diuretics can decrease the risk of pulmonary oedema in microgravity, there remains a potential risk of developing cardiogenic shock in HFrEF patients with inadequate total blood volume.

### 4.3 Implications for internal jugular vein thrombosis

Astronauts have been reported to experience internal jugular vein thrombosis (Auñón-Chancellor et al., 2020), and although the precise pathophysiology remains unclear, it can be hypothesized that elevated upper body venous pressure plays a role. Our simulations indicated an increase in right atrial pressure upon exposure to microgravity. Raised venous pressure has been associated with venous endothelial damage (Castro-Ferreira et al., 2018), which, in turn, is a risk factor for thrombosis development (Marshall-Goebel et al., 1985; Anderson and Spencer, 2003). This suggests a potential link between increased venous pressure, endothelial damage, and the occurrence of internal jugular vein thrombosis in astronauts. Further investigation is warranted to elucidate the exact pathophysiology of this disease in astronauts.

### 4.4 Limitations

The patient data obtained from the literature for HFrEF and HFpEF often involved individuals undergoing pharmaceutical treatment, resulting in simulations of controlled heart failure. However, it is reasonable to assume that individuals participating in space travel would adhere to their prescribed medication regimens.

HFrEF encompasses various cardiovascular hemodynamic effects influenced by aetiology, including increased neurohormonal activity (Packer, 1992), which was not accounted for in the model. HFpEF is now recognized as a heterogeneous disorder (Shah et al., 2014), and comprehensively incorporating all HFpEF-related parameters is beyond the scope of both the model and this study. In addition to increased ventricular elastance, HFpEF is associated with abnormal active relaxation of the heart (Kadry et al., 2020) which the current model cannot incorporate. Furthermore, the calculation of E/A ratios and the biphasic non-linear mechanism of left ventricular filling, often used for diagnosing and analysing HFpEF are not considered in this model.

The autonomic dysfunction accompanying HFrEF and HFpEF was left unchanged in reflex part of this mathematical model. We do however recognize that changes in autonomic reflexes may lead to alterations in orthostatic tolerance upon transitioning from supine to standing in individuals with heart failure. Signs of orthostatic intolerance may result in worse outcomes following exposure to microgravity environments, necessitating further investigation of this topic.

Heart failure is associated with a wide range of comorbidities, with a substantial proportion of patients attributing their clinical condition primarily to comorbidities (Loosen et al., 2022). Evaluating and simulating comorbidities associated with HFrEF and HFpEF fall outside the scope of this study. Further research should explore the simulation of “holistic” patients with both heart failure and associated comorbidities, considering the cumulative effect of comorbidities on heart failure following exposure to microgravity.

In the supine position in 1G of healthy astronauts, the ejection fractions were approximately 51%. While this value falls within the normal range according to most classifications, it could be considered mildly abnormal according to certain guidelines (Kanagala and Squire, 2020). This limitation may be attributed to the computational model’s exclusive consideration of the diastolic unstressed volume in cardiac fluid compartments, without accounting for the systolic unstressed volume in these compartments, as done in other hemodynamic models (Neal and Bassingthwaighte, 2007).

For the purpose of this research, the model’s simulation of microgravity entry has been simplified compared to the actual process experienced by space tourists. The model simulated a 10-s entry into microgravity, whereas reality involves a period of hypergravity lasting minutes before a near-instantaneous sudden transition to microgravity (Jackson, 2017). Future studies could investigate the effects of hypergravity on individuals with heart failure.

### 4.5 Future research

This study focused on elucidating safety concerns during space tourism, specifically during entry into microgravity. However, longer-term space travel, such as journeys to Mars, is associated with cardiac atrophy, alterations in pulmonary volumes and perfusion, electrical abnormalities, and other cardiovascular and hemodynamic changes (Schneider et al., 2016). Future research should explore the potential effects of extended space flight on individuals with cardiac pathologies.

Additionally, the feasibility of personalized modeling for individuals with underlying medical conditions entering microgravity should be examined to establish an individual’s risk profile for space tourism.

In conclusion, this study successfully simulated the effects of heart failure in humans going on a short-term spaceflight and determined the impact of these conditions on hemodynamic performance during microgravity entry. These findings might advance our understanding of the risks involved in space travel with heart failure and their implications for the success of space tourism missions.

## Data Availability

The raw data supporting the conclusion of this article will be made available by the authors, without undue reservation.
